# An Outlier Suppression and Adversarial Learning Model for Anomaly Detection in Multivariate Time Series

**DOI:** 10.3390/e27111151

**Published:** 2025-11-13

**Authors:** Wei Zhang, Ting Li, Ping He, Yuqing Yang, Shengrui Wang

**Affiliations:** 1 Department of Electrical Engineering, Hebei Vocational University of Technology and Engineering, Xingtai 054000, China; zhangwei@hevute.edu.cn; 2 School of Artificial Intelligence, Hebei University of Technology, Tianjin 300401, China; lt15632997337@163.com; 3 Technology Department, China Construction Bank, Beijing 100089, China; yqyang3130@163.com; 4 Faculty of Sciences, University of Sherbrooke, Sherbrooke, QC J1K 2R1, Canada; shengrui.wang@usherbrooke.ca

**Keywords:** multivariate time series, anomaly detection, outlier suppression transformer, adversarial learning

## Abstract

Multivariate time series anomaly detection is a critical task in modern engineering, with applications spanning environmental monitoring, network security, and industrial systems. While reconstruction-based methods have shown promise, they often suffer from overfitting and fail to adequately distinguish between normal and anomalous data, limiting their generalization capabilities. To address these challenges, we propose the AOST model, which integrates adversarial learning with an outlier suppression mechanism within a Transformer framework. The model introduces an outlier suppression attention mechanism to enhance the distinction between normal and anomalous data points, thereby improving sensitivity to deviations. Additionally, a dual-decoder generative adversarial architecture is employed to enforce consistent data distribution learning, enhancing robustness and generalization. A novel anomaly scoring strategy based on longitudinal differences further refines detection accuracy. Extensive experiments on three public datasets—SWaT, WADI, SMAP, and PSM—demonstrate the model’s superior performance, achieving an average F1 score of 88.74%, which surpasses existing state-of-the-art methods. These results underscore the effectiveness of AOST in advancing multivariate time series anomaly detection.

## 1. Introduction

Time series anomaly detection has emerged as a critical area of research in today’s increasingly intelligent society, demonstrating significant practical value across various domains, including environmental monitoring, network security, finance, manufacturing, and healthcare. In these fields, timely identification of anomalies is essential for enhancing security measures and mitigating potential economic losses [1,2,3]. For instance, in water treatment systems, effective anomaly detection is crucial for monitoring water quality and recognizing abnormal patterns, allowing for prompt intervention and corrective actions. However, the rarity of anomalies, coupled with the abundance of normal data, creates challenges in data labeling, rendering it both difficult and costly. Thus, unsupervised anomaly detection has become a promising avenue for research in this context [4,5].

Recent advancements in deep neural networks have significantly enhanced unsupervised learning capabilities [6]. Among these advancements, reconstruction-based unsupervised anomaly detection methods have gained considerable attention. These methods typically involve training an encoder on normal data to reconstruct sequences, subsequently applying the model to abnormal sequences to identify discrepancies. Anomalies are then detected based on predefined thresholds or other criteria. Notably, autoencoders (AEs) and their variants have emerged as prominent representatives of these methods, finding widespread application in unsupervised anomaly detection [7,8]. Recurrent neural networks (RNNs) have been integral to AEs for time series anomaly detection, as they effectively model sequential data and retain information from prior time steps [9]. Nevertheless, RNNs face several challenges, including the vanishing gradient problem, which impedes their performance in capturing long-term dependencies [10,11]. The introduction of long short-term memory (LSTM) networks addressed some of these challenges by alleviating the vanishing gradient problem and enhancing the ability to model long-range dependencies [12]. However, LSTMs are often hindered by high computational complexity, necessitating substantial resources and time. In light of these limitations, the Transformer architecture has been proposed as a viable alternative, offering enhanced global representation and long-distance correlation modeling capabilities, thereby effectively extracting relationships between time points [13]. This shift has led to notable advancements in the field [14,15].

Despite these progressions, several key challenges persist in the realm of time series anomaly detection. First, there is often insufficient distinction between normal and anomalous data points. The current differentiation information between normal and abnormal samples tends to be neither adequate nor explicit, leading to excessive generalization by models. This can result in anomalies generating minor reconstruction errors, which may cause misclassification as normal data. This issue is exacerbated when abnormal samples are incorrectly labeled as normal during training. Second, the reliance on training data composed solely of normal patterns renders the model susceptible to overfitting. In practical applications, the training dataset is frequently much smaller than the expansive and unobservable test dataset, heightening the risk of overfitting and diminishing the model’s generalization ability [16].

To address these challenges, we present a novel multivariate time series anomaly detection model, termed AOST (An Outlier Suppression Transformer), which integrates outlier suppression and adversarial learning within a Transformer framework. Specifically, we introduce an Outlier Suppression Attention (OSA) mechanism designed to enhance the distinction between normal and abnormal data points. By diminishing the emphasis on adjacent time steps, the model compels anomalies to produce larger reconstruction errors, thereby preventing the overgeneralization that can lead to misclassification. In addition, we incorporate an adversarial training strategy inspired by methodologies from natural language processing, which enables the model to learn from adversarial examples during training, thus alleviating overfitting and bolstering both robustness and generalization. Furthermore, we propose a novel anomaly scoring method based on longitudinal differences, quantifying the distributional gap between normal and abnormal data and offering a more comprehensive perspective for detection. Our contributions can be summarized as follows:

- We introduce AOST, a novel multivariate time series anomaly detection model that fuses adversarial learning with outlier suppression attention within a Transformer architecture.
- We develop OSA to amplify the distinctions between normal and anomalous samples and introduce a new anomaly scoring technique based on longitudinal differences, thereby enhancing detection capabilities.
- We implement adversarial training to mitigate overfitting, ultimately improving the model’s robustness and generalization.
- Comprehensive experiments conducted on three public datasets (SWaT, SMAP, and PSM) demonstrate that our model achieves an average F1 score of 90.74%, surpassing the best-performing method by 1.89% and showcasing advanced performance in the field.

## 2. Related Work

Time series anomaly detection is a burgeoning research area with significant application prospects, essential value, and extensive scholarly attention. Unsupervised learning techniques, particularly those applied to time series anomaly detection, have demonstrated commendable efficacy. Clustering represents a prevalent unsupervised learning strategy that organizes data into groups characterized by shared attributes, revealing underlying structures within the dataset. The core principle of employing clustering for anomaly detection involves grouping normal data points into coherent clusters while identifying abnormal points that form distinct, isolated clusters. For instance, the THOC model proposed by Shen et al. [17] leverages multi-scale time features extracted from the middle layer through a multi-layer clustering mechanism, identifying anomalies based on multi-layer distance metrics. Similarly, the ITAD model introduced by Shin et al. [18] utilizes clustering techniques for anomaly detection by decomposing tensors to reveal abnormal patterns.

In recent years, the advent of deep learning models has catalyzed continuous advancements in this field, particularly within the domain of unsupervised learning. A notable performance gap exists between traditional time series anomaly detection methods and those grounded in deep learning paradigms. Deep learning methodologies exhibit distinct advantages in capturing complex, high-dimensional temporal information inherent in multivariate time series data due to their robust model representation and automated feature extraction capabilities. For instance, Su et al. [9] introduced OmniAnomaly, a resilient model designed for detecting anomalies in multivariate time series data, utilizing techniques such as variational autoencoders and normal distribution modeling for efficient and accurate anomaly detection. Likewise, Li et al. [19] presented InterFusion, an unsupervised approach aimed at modeling dependencies in multidimensional time series data, which utilizes two random latent variables in conjunction with a variational autoencoder (VAE) to capture accurate patterns, further enhanced by a pre-filtering strategy. Zerveas et al. [20] introduced TransFram, a framework for representation learning of multivariate time series data based on a Transformer encoder, effectively employing the self-attention mechanism to uncover reliable long-range temporal correlations. While these methods adeptly leverage the intricate temporal dimensions of multivariate time series to extract features, they are constrained by their reliance on normal data exclusively during the training phase, rendering them susceptible to overfitting.

The integration of adversarial learning into time series anomaly detection has partially mitigated the susceptibility of models to overfitting during training. The USAD model, proposed by Audibert et al. [4], incorporates adversarial training methodologies rooted in sequence reconstruction tasks to enhance the model’s sensitivity to minor anomalies and overall performance. The TranAD model, introduced by Tuli et al. [21], employs an attention-based sequence encoder to facilitate rapid inferences, leveraging comprehensive temporal trend knowledge embedded in the data while utilizing meta-learning (MAML) for training with limited data. The AMA model, presented by Zhao et al. [22], integrates a novel deep memory autoencoder within a multivariate time series anomaly detection framework, incorporating a storage module to capture prototype feature vector information, thereby mitigating issues of model over-generalization. Furthermore, the Adformer model proposed by Zeng et al. [23] introduces an innovative architecture for detecting anomalies in multivariate time series, fusing adversarial learning with Transformer techniques. By employing a fused anomaly probability strategy and staged reconstruction errors as prior knowledge, this model effectively identifies abnormal behaviors in IoT sensor data. However, the aforementioned methods often lack a robust distinction between normal and abnormal data, leading to suboptimal capabilities in identifying anomalies.

Although recent studies have made progress in multivariate time-series anomaly detection, existing methods still exhibit significant limitations closely related to the challenges outlined in the Introduction, namely weak outlier discrimination, vulnerability to noise, and poor generalization. Clustering- and decomposition-based models (e.g., THOC, ITAD) often fail to capture long-range temporal dependencies; VAE-based approaches (e.g., OmniAnomaly, InterFusion) rely on smooth latent distributions that overlook subtle or abrupt anomalies; and Transformer-based frameworks (e.g., TranAD, Adformer) emphasize global correlations but lack mechanisms to suppress local outliers or stabilize attention learning.

To overcome these limitations, the proposed AOST framework integrates an Outlier Suppression Attention (OSA) mechanism and a dual adversarial autoencoder architecture, which jointly enhance anomaly discrimination and improve generalization in time series anomaly detection tasks.

## 3. Method Overview

### 3.1. Problem Statement

A multivariate time series is characterized by the simultaneous recording of multiple sensor variables at discrete time points, mathematically represented as $X=\left\{x_{1},\ldots ,x_{T}\right\}\in R^{T\times m}$, where *T* denotes the total length of the time series, *m* signifies the number of sensor variables, and $x_{t}\in R^{m}$ constitutes the *m*-dimensional vector corresponding to time point *t*. To effectively capture the temporal dependencies between the current time point and its preceding points, we introduce the concept of an input window $W_{t}$. As articulated in Equation (1), the time window encompasses a sequence of length *k* centered at time *t*:

(1) $$W_{t}=\left\{x_{t-k+1},\ldots ,x_{t-1},x_{t}\right\},$$

The process of multivariate time series anomaly detection can be delineated as follows: an input window $W_{t}$ is fed into the model, and the corresponding label $Y_{t}$ serves as the model’s output. The label $Y_{t}$ indicates the anomaly status at time *t*; specifically, $Y_{t}=1$ denotes the presence of an anomaly, while $Y_{t}=0$ signifies normalcy. In instances of abnormal behavior, the model must respond with both speed and precision to accurately identify the anomaly.

The proposed model employs a sliding window mechanism, whereby each time point is included in multiple overlapping windows. This results in the generation of several anomaly scores for each time point. In high-sensitivity scenarios, an alert may be triggered upon the detection of the first elevated score. Conversely, in contexts where precision is paramount, the average of the scores can be calculated to facilitate a more reliable identification of the anomaly’s location. This dual approach allows the model to effectively balance sensitivity and accuracy, optimizing its performance in diverse operational environments.

### 3.2. Model Architecture

**Figure 1:**
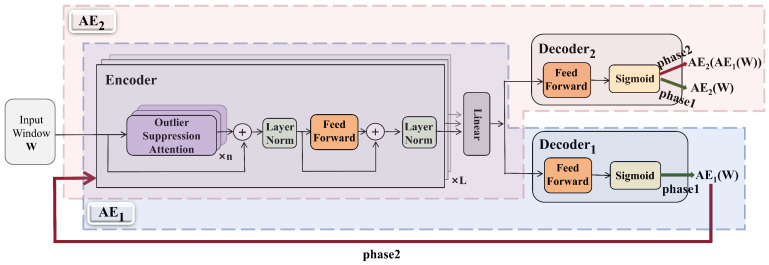
The proposed AOST model.

This section presents an overview of the proposed model, AOST, which is composed of an Encoder, ${\mathrm{Decoder}}_{1}$, and ${\mathrm{Decoder}}_{2}$. The initial phase involves feature learning facilitated by the Encoder, where the Outlier Suppression Attention (OSA) mechanism serves as the central component. Following this, the model employs ${\mathrm{Decoders}}_{1}$ and ${\mathrm{Decoders}}_{2}$ sequentially to implement adversarial learning. The overall architecture of the model is illustrated in Figure 1.

The design of dual autoencoders ($AE_{1}$ and $AE_{2}$) follows the theoretical motivation established in adversarial reconstruction frameworks such as USAD [4] and Adformer [23], which extend the principle of Generative Adversarial Networks into time-series anomaly detection. In these approaches, the interaction between two reconstruction networks helps to stabilize adversarial learning and improve generalization. This structured approach enables the model to effectively capture the intricate patterns within the multivariate time series data while enhancing its robustness and generalization capabilities through adversarial training. The synergy between the feature extraction and adversarial learning components is pivotal for achieving optimal performance in anomaly detection tasks.

#### 3.2.1. Encoder

The Encoder within the proposed model employs a Transformer encoder-based architecture, characterized by multiple layers incorporating Outlier Suppression Attention (OSA) and FeedForward components. This multi-layer stacked configuration is particularly effective for extracting complex correlations from deep and multi-level features present in the input data. The OSA mechanism plays a critical role by amplifying the distinctions between normal and abnormal data points, thereby enhancing the model’s sensitivity to anomalous deviations. A detailed description of OSA will be provided in Section 3.3.

The computation process of the Encoder is delineated in Equations (2) and (3):

(2) $$Z^{l}=\mathrm{LayerNorm}\left(\mathrm{OSA}\left(W^{l-1}\right)+W^{l-1}\right),$$

(3) $$Z^{l}=\mathrm{LayerNorm}\left(\mathrm{FeedForward}\left(Z^{l}\right)+Z^{l}\right),$$

where $W^{l}\in R^{k\times m}$ represents the input window, $Z^{l}\in R^{k\times m}$ denotes the hidden representation of the *l*-th layer, *l* indicates the layer number, *k* signifies the length of the input sequence, and *m* refers to the feature dimension. This architectural design allows the Encoder to effectively capture intricate patterns and relationships within the multivariate time series data, thereby laying a robust foundation for subsequent processing stages within the AOST model.

#### 3.2.2. ${\mathrm{Decoder}}_{1}$, ${\mathrm{Decoder}}_{2}$

Both ${\mathrm{Decoder}}_{1}$ and ${\mathrm{Decoder}}_{2}$ are structured as feed-forward layers, designed to perform dimensionality reduction from $R^{m}$ to $R^{d}$, as articulated in Equation (4):

(4) $${\mathrm{Decoder}}_{*}(\mathrm{Encoder}(W))=\mathrm{Sigmoid}(\mathrm{FeedForward}(\mathrm{Encoder}(W))),$$

As illustrated in Figure 1, the integration of the Encoder with ${\mathrm{Decoder}}_{1}$ forms the architecture denoted as $AE_{1}$, while the combination of the Encoder with ${\mathrm{Decoder}}_{2}$ constitutes $AE_{2}$. In the proposed model, $AE_{1}$ = Encoder + ${\mathrm{Decoder}}_{1}$ aims to learn the distribution of normal sequences through self-reconstruction. Its objective is to minimize the reconstruction loss and accurately reproduce normal patterns. In contrast, $AE_{2}$ = Encoder + ${\mathrm{Decoder}}_{2}$ is trained in opposition to $AE_{1}$. $AE_{2}$’s objective is to identify whether its input originates from genuine normal data or from $AE_{1}$’s reconstruction, thereby enforcing distributional discrimination. Notably, specific parameters within the $AE_{1}$ and $AE_{2}$ encoders are shared, as indicated in Equation (5):

(5) $$\begin{array}{cc} AE_{1}\left(W\right) & ={\mathrm{Decoder}}_{1}\left(\mathrm{Encoder}\left(W\right)\right),\\ AE_{2}\left(W\right) & ={\mathrm{Decoder}}_{2}\left(\mathrm{Encoder}\left(W\right)\right), \end{array}$$

This parameter-sharing mechanism enhances the model’s efficiency and consistency, allowing both decoders to leverage shared knowledge during the learning process. Consequently, this design facilitates improved performance in anomaly detection tasks, ensuring that both decoders can effectively interpret and process the features extracted by the Encoder.

### 3.3. Outlier Suppression Attention

Outlier Suppression Attention (OSA) has been developed to effectively address the challenge of distinguishing normal points from abnormal ones within multivariate time series data. Specifically, OSA employs both self-attention and Gaussian attention mechanisms to assess correlation differences across time steps, thereby enhancing the model’s capacity for anomaly detection. The components of OSA are detailed as follows:

(1) Self-Attention

In the self-attention mechanism, each point in the input sequence is compared with every other point. The self-attention distribution, denoted as S, is computed as follows:

(6) $$S=\mathrm{Softmax}\left(\frac{QK^{T}}{\sqrt{m}}\right),$$

where $Q=XW_{Q},\;K=XW_{K}$. $Q,K\in R^{m\times d}$ represent the query and key matrices, respectively. *X* represents the input sequence and *d* indicates the dimensionality of each vector. Each row in the self-attention distribution (i.e., $S_{i,\cdot }$) reflects the correlation of the *i*-th point with all other points in the sequence.

(2) Gaussian Attention

While the self-attention mechanism performs global (horizontal) comparisons across all time steps, such global operations may dilute the local temporal continuity that is essential for detecting abnormal fluctuations. To capture local coherence, we introduce a Gaussian attention mechanism [24], which focuses on local temporal correlations among neighboring time steps.

Let i,j∈{1,2,…,T} denote discrete indices of time steps in the input sequence *X*. The Gaussian attention between the *i*-th and *j*-th time points is defined using a Gaussian kernel based on the discrete distance |j−i|:

(7) $$G(|j-i|;\sigma_{i})=\frac{1}{\sqrt{2\pi }\sigma_{i}}\exp\left(-\frac{{\left|j-i\right|}^{2}}{2\sigma_{i}^{2}}\right),$$

where $\sigma_{i}$ is a learnable parameter representing the receptive width of the local temporal window centered at time step *i*. This Gaussian kernel assigns larger weights to temporally adjacent points and smaller weights to distant ones, thereby emphasizing local temporal continuity. The Gaussian attention distribution is subsequently rescaled to ensure numerical stability and comparability:

(8) $$G=\mathrm{Rescale}\left({\left[\frac{1}{\sqrt{2\pi }\sigma_{i}}\exp\left(-\frac{{\left|j-i\right|}^{2}}{2\sigma_{i}^{2}}\right)\right]}_{i,j\in \{1,\ldots ,n\}}\right),$$

**Figure 2:**
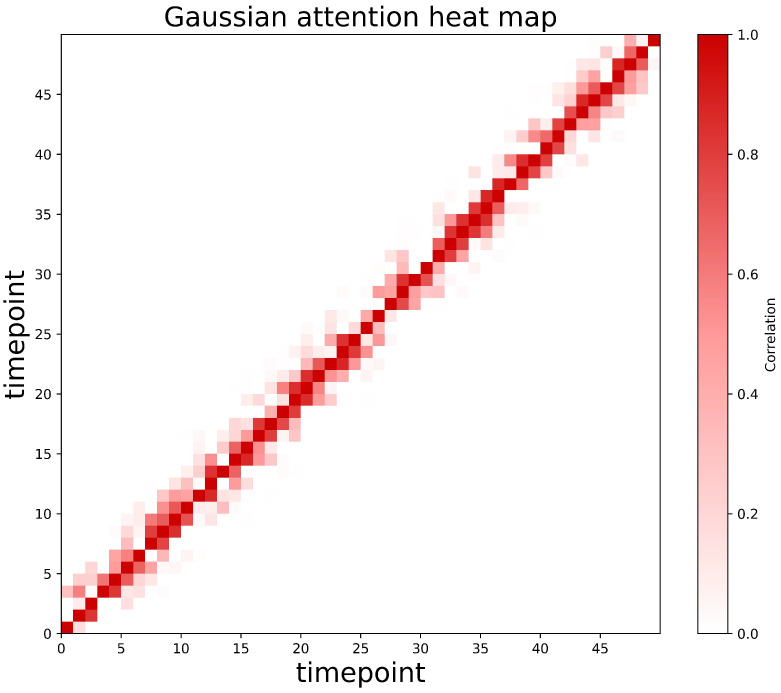
Gaussian attention heat map.

This Gaussian attention performs a local temporal comparison, measuring the internal smoothness of each time point with its neighbors, whereas self-attention captures global cross-time relationships. The joint use of both mechanisms enables the model to differentiate normal points, which maintain stable global correlations, from abnormal points, which primarily exhibit localized dependencies. As depicted in Figure 2, a heatmap illustrates the Gaussian attention for selected time series points. The heatmap on the right demonstrates the correlation, transitioning from red (indicating a higher correlation) to white (indicating a lower correlation) from top to bottom. The left side of the figure shows the Gaussian distribution obtained through Gaussian attention across 50 time points.

(3) Abnormal Longitudinal Difference

Abnormal points typically exhibit a strong correlation with nearby points but are less likely to correlate with the entire sequence. Conversely, normal points often maintain robust correlations with the full sequence. Given that the Gaussian attention distribution emphasizes relationships between points and their immediate neighbors, the distributions for abnormal points will generally exhibit similarity between their self-attention and Gaussian attention distributions. This similarity is not present for normal points. By leveraging the distinct characteristics of normal and abnormal points within the longitudinal distribution, we quantify the distance between the self-attention distribution and its corresponding Gaussian attention distribution at each time point, a metric we term the Abnormal Longitudinal Difference (ALD). This is calculated as follows:

(9) $$\mathrm{ALD}\left(G,S\right)={\left[\frac{1}{L}\sum_{l=1}^{L}\left(KL\left(G_{i,:}∥S_{i,:}\right)+KL\left(S_{i,:}∥G_{i,:}\right)\right)\right]}_{i=1,\ldots ,n},$$

The motivation behind the ALD is to measure the consistency between global and local temporal correlations captured by self-attention and Gaussian attention, respectively. Abnormal observations tend to correlate strongly with nearby points but weakly with the overall sequence, leading to similarity between their self-attention and Gaussian-attention distributions. Conversely, normal observations maintain more coherent long-range dependencies, resulting in greater divergence between the two distributions and thus larger ALD values. Since abnormal points typically yield smaller ALD values, this metric serves to effectively distinguish them from normal points. Training with ALD encourages each time point to attend to non-adjacent regions, thereby complicating the reconstruction of abnormal points.

(4) Multi-Head Implementation of OSA

**Figure 3:**
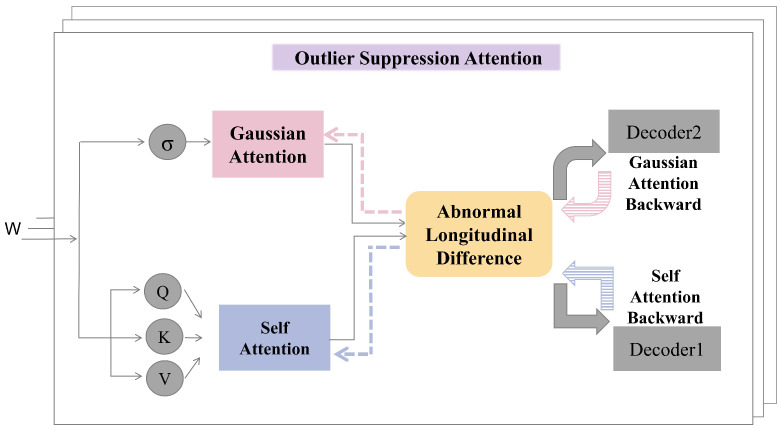
Outlier suppression attention.

To capitalize on the unique longitudinal differences of outliers, OSA is further extended through a multi-head design, as illustrated in Figure 3. This configuration allows the model to capture a variety of attention patterns across different representation subspaces, thereby enhancing its efficacy in suppressing outliers.

In summary, OSA integrates self-attention and Gaussian attention mechanisms to accentuate the longitudinal discrepancies between normal and abnormal points, thereby improving the performance of anomaly detection in time series data.

### 3.4. Model Training

The training process for AOST is conducted in two distinct stages. The initial stage focuses on OSA-based reconstruction, which emphasizes the differentiation between normal and abnormal points, thereby rendering the reconstruction of abnormal points more challenging. This heightened difficulty enhances the model’s ability to identify anomalies effectively.

In the subsequent stage, adversarial methods are employed to mitigate the risk of model overfitting. By integrating adversarial training techniques, the model is better equipped to generalize across diverse datasets and maintain robustness against variations in input data.

Collectively, these two stages work in concert to enhance the overall performance of the model, ensuring it achieves a higher degree of accuracy and reliability in anomaly detection tasks.

#### 3.4.1. Outlier Suppression Training

AOST employs reconstruction training that focuses on learning the latent features of normal data while minimizing reconstruction errors. To further amplify the distinction between normal and abnormal points, an Abnormal Longitudinal Difference (ALD) loss is integrated alongside the reconstruction loss. This dual-loss approach significantly reduces the reconstruction loss for normal points while maintaining higher reconstruction errors for abnormal points, thereby making the latter more challenging to reconstruct.

**Figure 4:**
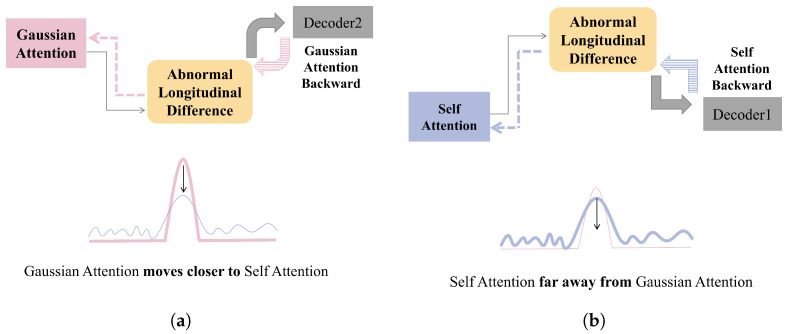
ALD training: (**a**) Gaussian Attention. (**b**) Self Attention.

However, a direct maximization of the ALD loss can result in a substantial decrease in the scale parameter of the Gaussian kernel [25], which may compel the Gaussian attention mechanism to concentrate solely on a single point. To better regulate the behavior of Gaussian attention, the ALD loss is initially assigned a low value. This strategy enables the Gaussian attention to closely approximate the self-attention distribution of the original sequence and to adapt effectively to diverse time-series patterns (refer to Figure 4a). As training progresses, the ALD loss is gradually increased, which encourages the self-attention mechanism to allocate greater attention to non-adjacent points within the sequence (as illustrated in Figure 4b) [24].

Consequently, the loss function for the first stage, which incorporates both reconstruction loss and ALD, is formulated as follows:

(10) $$L_{AE_{1}\_S_{1}}={∥W-AE_{1}\left(W\right)∥}_{2}+\lambda \times {∥ALD\left(G,S\right)∥}_{2},$$

(11) $$L_{AE_{2}\_S_{1}}={∥W-AE_{2}\left(W\right)∥}_{2}-\lambda \times {∥ALD\left(G,S\right)∥}_{2},$$

where ∥•∥ denotes the $L_{2}$ norm, and λ is a hyperparameter with λ>0. This structured approach to loss function design enables the model to effectively enhance its capacity for distinguishing between normal and abnormal data points during the training process.

#### 3.4.2. Adversarial Training

To mitigate the risk of overfitting, AOST employs an adversarial training strategy. Within this framework, the generative network aims to produce samples indistinguishable from real data, while the discriminative network endeavors to ascertain whether a given sample is authentic or generated. These two objectives are trained in an alternating fashion. After sufficient iterations, the generator becomes proficient at approximating the latent multivariate distribution of the training sequences, which effectively captures the normal operational state of the system. Concurrently, the discriminator enhances its ability to differentiate between authentic and synthesized data [26].

As depicted in Figure 1, $AE_{1}$, which consists of the Encoder and ${\mathrm{Decoder}}_{1}$, functions as the generative network, while $AE_{2}$, comprising the Encoder and ${\mathrm{Decoder}}_{2}$, serves as the discriminative network. The training objective for $AE_{1}$ is to refine its sequence reconstruction capabilities to such an extent that $AE_{2}$ is unable to determine whether the input data is derived from real samples or reconstructed by $AE_{1}$. Conversely, $AE_{2}$ is tasked with identifying whether the input data originates from genuine samples or is a reconstruction from $AE_{1}$. These objectives are formally articulated in Equations (12) and (13):

(12) $$L_{AE_{1}\_S_{2}}={∥W-AE_{2}\left(AE_{1}\left(W\right)\right)∥}_{2},$$

(13) $$L_{AE_{2}\_S_{2}}=-{∥W-AE_{2}\left(AE_{1}\left(W\right)\right)∥}_{2},$$

This adversarial training framework not only enhances the robustness of the model but also ensures that it can effectively generalize across different datasets, thereby improving its performance in anomaly detection tasks. Although the training process draws inspiration from the adversarial principle of GANs [26], the objective of AOST is not data generation but the refinement of normal reconstruction distribution. $AE_{1}$ and $AE_{2}$ form a dual-decoder adversarial pair that jointly optimize reconstruction consistency and discrimination, thus preventing overfitting and improving robustness.

#### 3.4.3. Total Training Loss

During the training phase, both $AE_{1}$ and $AE_{2}$ are guided by dual objectives. For $AE_{1}$, the primary goal is to minimize the reconstruction error between $AE_{1}\left(W\right)$ and *W*, thereby enhancing its reconstruction capabilities. By effectively reducing the ALD loss, the Gaussian attention mechanism increasingly aligns with the self-attention distribution, allowing for better adaptation to varying time-series patterns. Additionally, $AE_{1}$ strives to minimize the reconstruction error between $AE_{2}\left(AE_{1}\left(W\right)\right)$ and *W*, which further augments its data generation capabilities. The specific formulation of these objectives is presented in Equation (14).

Conversely, $AE_{2}$ aims to minimize the reconstruction error between $AE_{2}\left(W\right)$ and *W*, which contributes to its reconstruction performance. By maximizing the ALD loss, the model directs greater attention to non-adjacent points within the sequence, thus complicating the reconstruction of outliers. Furthermore, $AE_{2}$ endeavors to maximize the reconstruction error between $AE_{2}\left(AE_{1}\left(W\right)\right)$ and *W*, enhancing its discrimination capabilities, as indicated in Equation (15). The relative weights of each component within these dual tasks evolve dynamically throughout the training process.

In summary, the total training loss for the proposed model is expressed as follows:

(14) $$L_{AE_{1}}=\frac{1}{n}\left(L_{AE_{1}\_S_{1}}\right)+\left(1-\frac{1}{n}\right)\left(L_{AE_{1}\_S_{2}}\right),$$

(15) $$L_{AE_{2}}=\frac{1}{n}\left(L_{AE_{2}\_S_{1}}\right)+\left(1-\frac{1}{n}\right)\left(L_{AE_{2}\_S_{2}}\right),$$

(16) $$L=L_{AE_{1}}+L_{AE_{2}},$$

where *n* denotes the number of training iterations. In the early stage of training (small *n*), a larger weight on the OSA term encourages the model to effectively suppress outliers and learn representative normal patterns. In the later stage (large *n*), the training gradually shifts toward adversarial reconstruction, which allows $AE_{1}$ and $AE_{2}$ to refine temporal dependencies and improve robustness. This training process is further elucidated in Algorithm 1, which outlines the AOST training procedure.

**Algorithm 1:** The AOST training algorithm

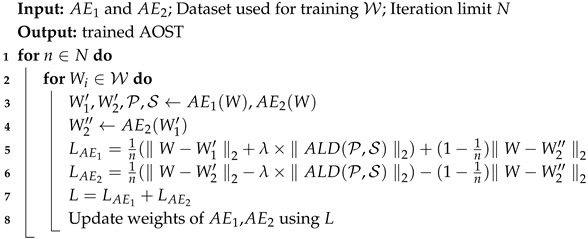



### 3.5. Anomaly Detection

For the purpose of anomaly detection, we introduce a novel anomaly scoring strategy that leverages abnormal longitudinal differences. This approach integrates the discrepancies between self-attention and Gaussian distributions, along with the model’s reconstruction residuals, thereby offering a more nuanced method for differentiating between normal and abnormal data. In practice, any data point with an anomaly score exceeding a predetermined threshold is classified as anomalous. The anomaly score is defined in Equation (17):

(17) $$\begin{array}{c} A\left(\hat{W}\right)=\alpha \left(\mathrm{Softmax}\left(-ALD\left(P,S\right)⊙∥\hat{W}-AE_{1}\left(\hat{W}\right)∥\right)\right)+\\ \beta \left(\mathrm{Softmax}\left(-ALD\left(P^{\prime },S^{\prime }\right)⊙∥\hat{W}-AE_{2}\left(AE_{1}\left(\hat{W}\right)\right)∥\right)\right), \end{array}$$

where ⊙ denotes the dot product. The variables P and S represent the Gaussian attention and self-attention distributions of the test sequence $\hat{W}$, as computed by the Encoder. Similarly, $P^{\prime }$ and $S^{\prime }$ signify the Gaussian attention and self-attention distributions of $AE_{1}\left(\hat{W}\right)$. The negative sign before ALD(P,S) indicates that larger attention discrepancies contribute positively to the overall anomaly score. This transformation ensures that abnormal patterns, which show greater inconsistency between P and S, receive higher anomaly scores, aligning the scoring function with the degree of abnormality. The parameters α and β (where α+β=1) are used to balance the trade-off between false positives and true positives.

When α>β, both the number of true positives and false positives diminish, indicating a low-sensitivity detection scenario. Conversely, when α<β, the number of true positives increases, albeit with a corresponding rise in false positives, resulting in a high-sensitivity detection scenario. By adjusting the values of α and β, the sensitivity of anomaly detection can be tailored to suit various application contexts. Algorithm 2 provides a comprehensive overview of the anomaly detection process implemented by the model.

**Algorithm 2:** The AOST test algorithm

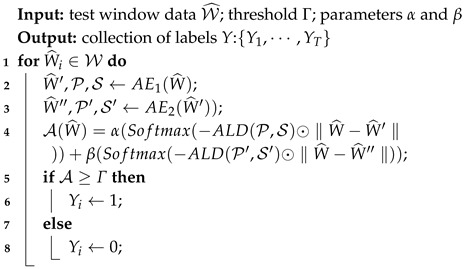



## 4. Experiments

### 4.1. Datasets

The experiments were conducted using three publicly available datasets: the Secure Water Treatment (SWaT) dataset [27,28], the Water Distribution (WADI), the Soil Moisture Active Passive (SMAP) dataset [29], and the Pooled Server Metrics (PSM) dataset [30].

**Figure 5:**
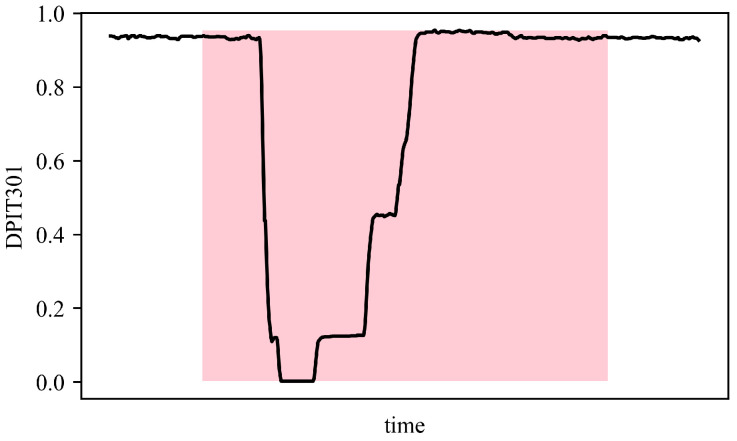
Display of an anomaly example.

The experiments were conducted on four publicly available datasets: SWaT, WADI, SMAP, and PSM datasets. SWaT and WADI are operational water-treatment testbeds widely used for industrial anomaly-detection research. SWaT records normal and attack periods in a small-scale plant, while WADI extends to a larger distribution network with more complex sensor interactions. Typical anomalies in these datasets include abrupt changes in flow, valve malfunctions, or inconsistent tank-level readings. SMAP contains environmental measurements such as soil moisture and temperature from NASA satellites, where anomalies often reflect sensor faults or transmission errors. PSM is collected from eBay’s production servers and captures anomalies caused by software or hardware faults. These datasets together cover diverse domains and anomaly patterns, providing a comprehensive evaluation basis for the proposed AOST model.Table 1 summarizes the characteristics of the datasets utilized in our experiments. An example of an anomaly is illustrated in Figure 5, where the pink-shaded regions denote abnormal behaviors, while the remaining parts correspond to normal patterns.

This comprehensive overview of the datasets underscores their relevance and applicability in the context of anomaly detection research.

### 4.2. Evaluation Metric

In this experiment, a standardized methodology is employed to evaluate the performance of the AOST model. The key evaluation metrics utilized are Precision (*P*), Recall (*R*), and F1-score ($F_{1}$), as defined in Equation (18). Specifically, TP denotes the number of positive samples accurately predicted by the model, FN represents the count of positive samples incorrectly classified as negative, and FP indicates the number of negative samples incorrectly predicted as positive. Additionally, TN signifies the number of negative samples correctly identified by the model. In the context of anomaly detection, anomalies are treated as a minority class and designated as the positive class, while the normal instances, representing the majority class, are classified as the negative class.

The evaluation metrics are expressed mathematically as follows:

(18) $$P=\frac{TP}{TP+FP},\;R=\frac{TP}{TP+FN},\;F_{1}=2\times \frac{P\times R}{P+R},$$

Anomalies frequently occur within designated time frames known as abnormal periods. A widely accepted adjustment strategy proposed by researchers [31] stipulates that if any time point within a continuous segment of abnormality is detected, it is assumed that all anomalies within that segment have been correctly identified. This strategy is deemed reasonable based on empirical observations, as in practical applications, the detection of abnormal time points typically triggers alarms and draws the attention of inspection personnel for a specified duration. Consequently, our model adopts this adjustment strategy to enhance its effectiveness in anomaly detection scenarios.

### 4.3. Experimental Details

All experiments were conducted using the PyTorch1.9.0 framework on an NVIDIA RTX 3090 GPU server. The input data underwent normalization and was subsequently divided into sliding windows of length k=12. The architecture of the encoder comprises three layers, each equipped with eight attention heads. The model utilized a batch size of 1024 and was optimized with a learning rate set at 0.0001. The ALD parameter was configured to λ=4, while the parameters for anomaly scoring were established with α=β=0.5.

Training was carried out for 100 epochs, employing the Adam optimizer [32] along with an early stopping strategy, which was implemented with a patience of 8 epochs to mitigate the risk of overfitting. For the purpose of anomaly detection, the threshold Γ was determined based on the validation dataset; any test sample with an anomaly score exceeding this threshold was classified as an anomaly. This experimental setup ensured a robust evaluation of the AOST model’s performance in detecting anomalies across the datasets.

### 4.4. Experimental Results and Comparative Analysis

Our model was subjected to a comprehensive comparison with eight baseline models: Adformer, TranAD, InterFusion, TransFram, USAD, ITAD, THOC, and OmniAnomaly. Among them, THOC and ITAD detect anomalies through hierarchical clustering and tensor decomposition, respectively. OmniAnomaly and InterFusion employ variational autoencoders to model probabilistic latent spaces. TransFram and TranAD adopt Transformer-based architectures to learn long-range dependencies. USAD and Adformer introduce adversarial reconstruction frameworks enhancing robustness.

The experimental results are presented in Table 2, where the highest performance in terms of the F1 score is highlighted in bold, while the second-best performance is underlined. Each experiment was repeated three times, and the reported results represent the average performance across runs.

From the results presented in Table 2, several key conclusions can be drawn. First, our model achieves the highest F1 score across the SWaT, WADI and SMAP datasets, and it attains the second-best result on the PSM dataset, with a marginal difference of merely 0.05% from the leading result. Notably, the F1 scores for both the SMAP and PSM datasets exceed 90%. Moreover, on the WADI dataset, the proposed AOST model outperforms the second-best method (Adformer) by 1.23%. This improvement further validates the robustness and generalization ability of AOST under complex multivariate temporal dependencies. Second, unlike THOC and ITAD, our model effectively incorporates the learning of complex temporal information inherent in multivariate time series data. Additionally, when compared to OmniAnomaly, InterFusion, and TransFram, the incorporation of an adversarial learning strategy in our model leads to substantial performance enhancements. Furthermore, in contrast to USAD, TranAD, and Adformer, our model establishes a clear distinction between normal and abnormal samples. With the exception of Adformer, which surpasses our model by 0.05% on the PSM dataset, the other baseline models exhibit inferior results. In practical model selection, specific requirements should be taken into account. When optimizing the F1 score is the priority, our proposed model is the preferred choice. When minimizing missed detections is more critical, recall becomes the primary consideration, as exemplified by InterFusion. Conversely, when minimizing false alarms is essential, precision should be prioritized, as demonstrated by models such as USAD and Adformer.

**Figure 6:**
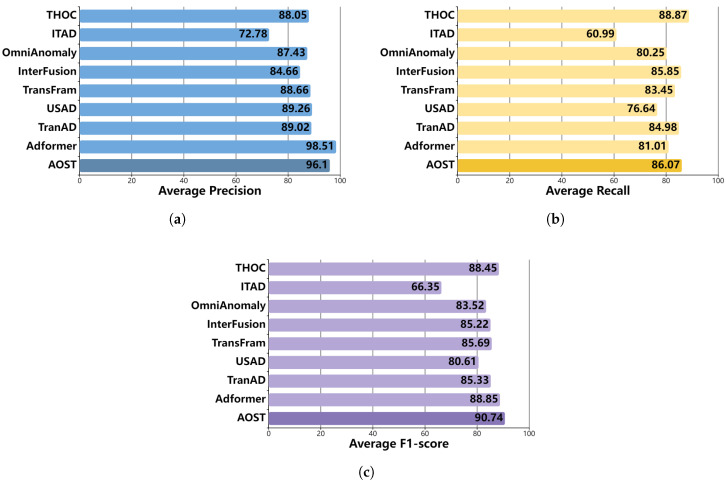
Average performance comparison of models. (**a**) Average Precision. (**b**) Average Recall. (**c**) Average F1-score.

Figure 6 illustrates the average performance of all evaluated methods across the three public datasets. Our model achieves an average F1 score exceeding 90%, reflecting optimal performance. Remarkably, this score is 1.89% higher than that of the best-performing method. Moreover, our model demonstrates a balanced performance regarding the trade-offs between precision and recall, with both average precision and average recall reaching the second-best level. Although Adformer achieves the highest average precision score, its average recall ranks sixth. Conversely, THOC records the highest average recall score, while its average precision also ranks sixth. In summary, our model consistently delivers optimal results across various metrics.

### 4.5. Anomaly Detection Visualization

**Figure 7:**
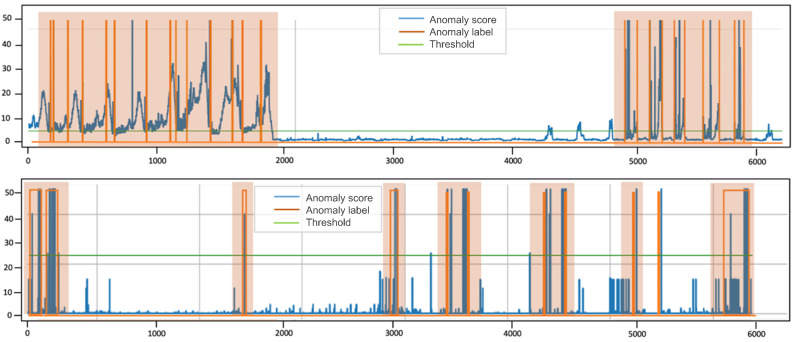
Visualization of anomaly detection.

An intuitive evaluation of the AOST model’s performance in anomaly detection is illustrated by visualizing the anomaly scores from selected test sets of the SMAP and PSM datasets, as depicted in Figure 7.

The highlighted region in the figure corresponds to the time frame characterized by a heightened occurrence of anomalies, during which the AOST model generates significantly higher anomaly scores compared to other periods. Conversely, lower scores are observed during normal operational periods. The threshold effectively delineates normal from abnormal intervals, thereby indicating a successful outcome in the model’s anomaly detection capabilities. This visualization underscores the model’s effectiveness in identifying deviations from expected behavior within the time series data. Such performance benefits from the model’s adversarial structure and abnormal longitudinal difference, which jointly enhance sensitivity to abnormal temporal dependencies while maintaining robustness against noise.

### 4.6. Ablation Study

To gain deeper insights into the efficacy of the model, ablation experiments were conducted to evaluate the impact of each individual component. The results of these experiments are summarized in Table 3.

The data presented in Table 3 indicate that when only adversarial learning is incorporated, the model achieves optimal precision (*P*), suggesting a high precision rate but a comparatively low recall rate (*R*). This outcome reflects the model’s limited capacity to identify abnormal data while demonstrating a stronger tendency to fit normal data. Conversely, the model that utilizes only Outlier Suppression Attention (OSA) exhibits the highest recall, indicating an enhanced ability to detect anomalies but a reduced capacity to accurately model normal data.

A significant improvement in overall performance is observed when both OSA and adversarial learning are employed simultaneously. The ablation experiments further confirm that OSA effectively amplifies the distinctions between normal and abnormal samples, thereby making abnormal points more challenging to reconstruct and enhancing the model’s anomaly detection capabilities. Concurrently, the integration of adversarial learning strategies serves to mitigate the risk of model overfitting. Ultimately, these enhancements contribute to improved generalization performance and robustness of the AOST model.

### 4.7. Parameter Analysis

#### 4.7.1. Encoder Layers Analysis

The number of encoder layers significantly influences the model’s runtime and overall performance. Consequently, we conducted an analysis to investigate how different configurations of encoder layers affect both runtime and F1 score. The results of this analysis are summarized in Table 4.

As evidenced by Table 4, increasing the number of encoder layers is associated with an enhancement in the F1 score. This improvement can be attributed to the greater expressive power of features when more encoder layers are utilized. A notable performance gain is observed with the two-layer encoder in comparison to the one-layer encoder, particularly on the SWaT dataset, where the F1 score increased by 35.6%, alongside a 20% increase in training time. However, when comparing the performance gains and training time of the three-layer encoder to the two-layer encoder, it is noted that on the SWaT dataset, the F1 score improved by only 0.22%, while the training time escalated by 66.7%. For the SMAP dataset, the F1 score also increased by 0.22%, with a 46% rise in training time. Similarly, on the PSM dataset, the F1 score showed a marginal increase of 0.11%, but the training time rose by 50%.

The model utilizing a three-layer encoder provides only a minimal enhancement in the F1 score relative to the two-layer encoder, yet it incurs a considerable increase in training time. Based on these findings, we have opted for a two-layer encoder to strike an optimal balance between accuracy and runtime across all datasets.

#### 4.7.2. Window Lengths Parameter and ALD Parameter Analysis

**Figure 8:**
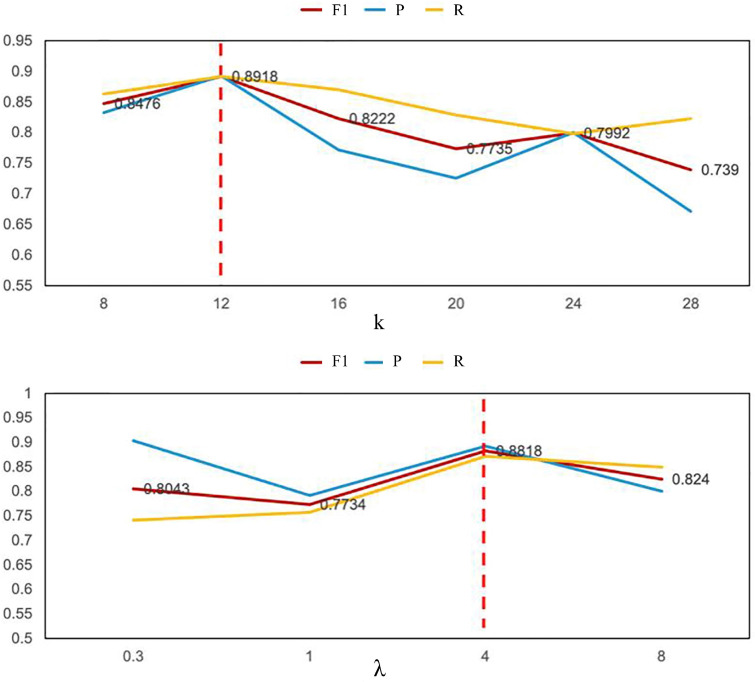
Parameter *k* and λ settings and results.

A sensitivity analysis was conducted to evaluate the model’s performance under varying parameters for sliding window length *k* and the ALD parameter λ. Figure 8 illustrates the trend of evaluation metrics as *k* and λ increase. The optimal F1 score is achieved with a sliding window length of k=12. A window that is too small may impede the model’s capacity to capture essential temporal patterns, whereas an excessively large window can lead to overfitting and result in unstable performance. Therefore, selecting an appropriate value for *k* is critical, as corroborated by the experimental findings.

Additionally, we investigated the influence of λ on model performance, determining that λ=4 yields the most favorable results. A very small λ does not sufficiently encourage the model to concentrate on non-adjacent sequences, effectively nullifying the impact of ALD. Conversely, an excessively large λ may lead to an overemphasis on non-adjacent points, thereby diminishing the model’s ability to accurately reconstruct normal data. Consequently, selecting an appropriate value for λ is essential to ensure the effectiveness of OSA.

#### 4.7.3. Abnormal Scoring Parameter Analysis

**Figure 9:**
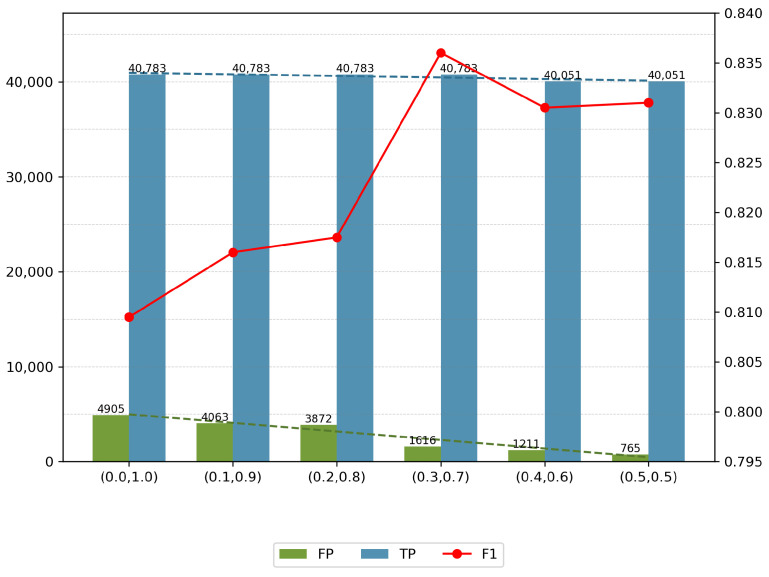
Parameter α and β settings and results.

This section examines the impact of the parameters α and β (as presented in Equation (17)) on the sensitivity of anomaly detection within the scoring framework. An increase in α enhances the emphasis on $AE_{1}$ during reconstruction, whereas a higher β prioritizes the contribution of $AE_{2}$. Notably, in certain scenarios, the sensitivity of anomaly detection can be adjusted without the need to retrain the model. By varying the values of α and β, we explored their effects on false positives (FP), true positives (TP), and the F1 score within the SWaT dataset, as illustrated in Figure 9.

From Figure 9, it is evident that both increasing and decreasing the parameters can significantly reduce the number of false positives, achieving an 85% reduction when transitioning from α=0.0 to α=0.5, while only resulting in a slight decrease of 1.7% in true positives over the same range. Consequently, the adjustment of α and β allows for parameterization of the model’s sensitivity, effectively aligning it with the requirements of specific operational environments.

The proposed model accommodates varying sensitivity levels, enabling detection processes to meet the diverse needs of regulatory teams. A lower sensitivity setting is particularly suitable for managers focused on minimizing false positives and generating alerts solely for significant anomalies. Conversely, a higher sensitivity setting is advantageous for technicians who require comprehensive event detection to avoid overlooking any unusual occurrences.

## 5. Conclusions

This study introduces the AOST model, which integrates the outlier suppression Transformer method with adversarial learning to enhance multivariate time series anomaly detection. By leveraging temporal differences of outliers, the model effectively distinguishes between normal and abnormal data, as demonstrated by its reduced reconstruction of anomalies. Experimental results validate the model’s robustness and generalization, with significant improvements in detection accuracy compared to state-of-the-art methods. These findings underscore the potential of AOST for applications in diverse anomaly detection scenarios, while future research could explore its adaptability to other domains and refine its scalability.

In future work, we plan to focus on subtle and hard-to-detect anomalies that exhibit weak temporal deviations or occur under complex multi-variable correlations. Such anomalies are common in real-world industrial and environmental systems but remain challenging for most current detection models.

## Figures and Tables

**Table 1:** Dataset Statistics.

Dataset	Training Samples	Testing Samples	Dimensions	Anomaly Rate (%)
SWaT	496,800	449,919	51	11.98
WADI	1,048,571	172,801	123	5.99
SMAP	135,183	427,617	25	13.11
PSM	132,481	87,841	25	27.8

**Table 2:** Comparison of Experimental Results with Baseline Models (F1 Score). The WADI dataset is additionally included for comprehensive evaluation.

Method	SWaT (%)			SMAP (%)			PSM (%)			WADI (%)		
	**P**	**R**	**F1**	**P**	**R**	**F1**	**P**	**R**	**F1**	**P**	**R**	**F1**
THOC	83.94	**86.36**	85.13	92.06	89.34	**90.68**	88.14	**90.99**	89.54	68.13	74.26	71.06
ITAD	63.13	52.08	57.08	82.42	66.89	73.85	72.80	64.02	68.13	56.62	48.38	52.18
OmniAnomaly	81.42	84.30	82.83	92.49	81.99	86.92	88.39	74.46	80.83	75.94	62.80	68.75
InterFusion	80.59	85.58	83.01	**98.77**	88.52	89.14	83.61	83.45	83.52	83.44	**86.53**	84.96
TransFram	92.47	75.88	83.36	85.36	87.48	86.41	88.14	86.99	87.56	81.16	83.69	82.41
USAD	98.70	74.02	84.60	76.97	98.31	86.34	92.10	57.60	70.90	72.88	80.58	76.54
TranAD	97.60	69.97	81.51	80.43	**99.99**	89.15	96.44	87.37	91.68	84.89	82.96	83.91
Adformer	**98.90**	76.18	86.07	97.30	80.37	88.03	**99.34**	86.47	**92.46**	90.25	81.24	85.51
AOST	96.92	80.63	**88.02**	94.86	88.93	**91.80**	96.53	88.64	92.41	**90.81**	83.01	**86.73**

**Table 3:** Ablation Results. In each row of the table, × indicates that the technique (sunch as Outlier Suppression or Adversarial Learning) was not used in the ablation experiment, and ✓ indicates that the technique was used.

Outlier Suppression	Adversarial Learning	P (%)	R (%)	F1 (%)
×	✓	99.44	68.58	81.17
✓	×	64.12	81.58	71.78
✓	✓	96.92	80.63	88.02

**Table 4:** Encoder Layers Analysis. The bold represent the best performence.

Encoder Layers	SWaT		SMAP		PSM	
	**Time (min)**	**F1 (%)**	**Time (min)**	**F1 (%)**	**Time (min)**	**F1 (%)**
1	10	64.9	11	78.8	9	81.0
2	12	88.0	13	**91.8**	10	92.4
3	20	**88.2**	19	92.0	15	**92.5**

## Data Availability

All datasets used in this study are available upon request from the corresponding author.
